# Stability-Indicating RP-HPLC Method for the Simultaneous Determination of Prazosin, Terazosin, and Doxazosin in Pharmaceutical Formulations

**DOI:** 10.3797/scipharm.1204-15

**Published:** 2012-05-22

**Authors:** Alankar Shrivastava, Vipin B. Gupta

**Affiliations:** B. R. Nahata College of Pharmacy, Mhow-Neemuch Road, Research Scholar, Jodhpur National University, Jodhpur, Rajasthan, India.

**Keywords:** Prazosin, Terazosin, Doxazosin, α_1_ Adrenoreceptor blockers, Stress degradation, Chromatography, Method validation

## Abstract

The current study was carried out with an attempt to separate similarly structured title drugs by liquid chromatography. Spectrophotometric techniques were generally insufficient under these conditions because of the spectral overlapping of drugs with similar functional groups. The pharmaceutical drugs prazosin, terazosin, and doxazosin contain the same parent quinazoline nucleus, thus making it especially difficult to separate the former two drugs because of their very similar structures. A simple and sensitive method for the routine determination of these drugs in pharmaceutical formulations was attempted. We found that the mobile phase consisting of A: ACN–diethylamine (0.05 ml), B: methanol, and C: 10 mM Ammonium acetate separated these drugs effectively. Separations were carried out on a new Kromasil C18 column (250 × 4.6 mm, 5.0 μm) at 254 nm wavelength. The calibration curve was found to be linear in the range of 2–500 μg/ml. The stated method was then validated in terms of specificity, linearity, precision, and accuracy. Additionally, the proposed method reduced the duration of the analysis.

## Introduction

High Pressure Liquid Chromatography (HPLC) is a well-known and widely used analytical technique which is prevalent throughout the pharmaceutical industry as a research tool for the estimation of impurities in drug substances and drug products [1]. As a result of significant technological improvements in instrumentation and column packings, high performance liquid chromatography (HPLC) has emerged as the preferred method for the separation and quantitative analysis of a wide range of samples [2].

Stability testing forms an important part of the process of drug product development. The purpose of stability testing is to provide evidence on how the quality of a drug substance varies with time under the influence of a variety of environmental factors such as temperature, humidity, and light, which enables recommendation of storage conditions, retest periods, and establishing shelf life [1]. An ideal stability-indicating chromatographic method should estimate the drug and also be able to resolve the drug from its degradation products [3].

The stability-indicating assay is a method that is employed for the analysis of stability samples in the pharmaceutical industry. It is also required that analytical methods should be validated to demonstrate that impurities unique to the new drug substance do not interfere with or are separated from specified and unspecified degradation products in the drug product [4].

The condition known as benign prostatic hyperplasia may be defined as a benign enlargement of the prostate gland resulting from a proliferation of both benign epithelial and stromal elements. It might also be defined clinically as a constellation of lower urinary tract symptoms (LUTSs) in aging men [5].

Traditionally, the pathogenesis of benign prostatic hyperplasia (BPH) and LUTS was explained as the interaction between hormonal and genetic factors [6]. The medical treatment of LUTS/BPH mainly involves the inhibition of the enzyme 5α-reductase to reduce prostate size and α_1_-adrenoceptor antagonists. The latter are more frequently used as they reduce LUTS more effectively than the 5α-reductase inhibitors in most patients [7]. Alpha-1 blockers are the first option for the medical treatment of LUTS caused by BPH [8]. Their effects are prompt, are well-tolerated, and remain efficacious in the long-term [9]. α-Adrenoreceptor antagonists are frequently used to treat patients with LUTS and benign prostatic enlargement because of their significant effect on storage (of urine) and voiding symptoms, quality of life, flow rate, and postvoid residual urine volume [10].

Potentiometric titration is an official method in British Pharmacopoeia [11] and Indian Pharmacopoeia [12] with 0.1 M perchloric acid as a titrant for the determination of prazosin. Radioreceptor assay [13] and voltametric [14] estimation methods are also found. The titrimetry method for the determination of terazosin by using 0.1 N NaOH, is official in European pharmacopoeia [15]. Other literature about the potentiometric estimation of terazosin [16] using 0.1 N perchloric acid and 0.1 N silver nitrate solution as the titrant, and by using a modified glass calomel electrode and glass silver electrode pair system, respectively, is also available. For doxazosin, two voltametric [17, 18] and polarographic [19] methods are reported in the current literature. Six spectrophotometry [14, 17, 20–23], four TLC [16, 25], twenty-six HPLC [13, 15, 16, 25–35], one HPTLC [36], and one HILIC-MS/MS [35] were methods in various matrixes that were also found during the literature survey.

**Fig. 1:**
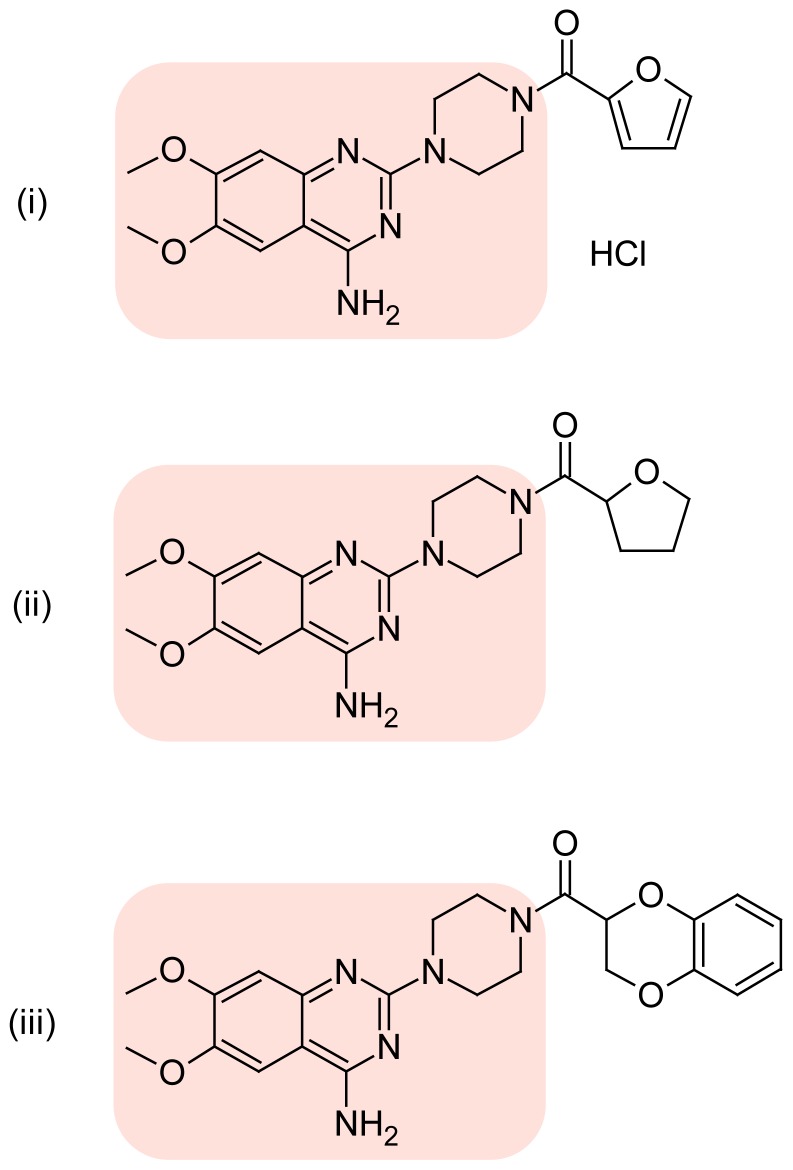
Chemical structures of three alpha one adrenoreceptor blockers. (i) Prazosin hydrochloride, (ii) Terazosin and (iii) Doxazosin [6,7-dimethoxy-2-(piperazin-1-yl)quinazolin-4-amine nucleus highlighted]

However, the common mobile phase in which drugs under investigation may separate, is reported by Bakshi et al [37]. In this method terazosin, prazosin, and doxazosin were run separately under same chromatographic conditions. The retention time of the terazosin and prazosin is close enough for them to merge if simultaneous determination is attempted for the determination of these structurally similar drugs. Prazosin, terazosin, and doxazosin contain same parent quinazoline (Figure 1) nucleus, and thus it is especially difficult to separate the former two drugs. Applications of conventional UV spectrophotometric methods are limited due to the spectral overlap throughout the wavelength. So, the gradient HPLC method can be used to overcome this problem. The proposed method reduced the duration of the analysis. This theory forms the basis of our study. Thus a simple and sensitive method for the routine determination of these pharmaceuticals in formulations was attempted. The proposed method allows the simultaneous determination of the three α_1_-adrenoreceptor blockers in this study without the need to carry out previous separation. The advantages of this procedure are simple chromatographic conditions and sparing the solvent.

There is no doubt on the fact that the spectroscopic methods [14, 17, 20–23] mentioned in the above texts are rapid and far more economical than chromatographic methods, but their destructive nature and lack of sensitivity is a huge disadvantage. The HPLC-EMS [32], UPLC-MS/MS [33], and HILIC-MS/MS [36] methods found during the literature survey have good sensitivity and are useful in the investigation of biological samples. Increased sensitivity of such types of methods is mostly compromised with complicated instrumentations, procedures, and mobile phases. Moreover, stability-indicating procedures do not require such complicated procedures, except when the structure elucidation of degradant products is an essential part of the method development.

## Experimental

### Reagents and chemicals

Doxazosin was kindly gifted by Dr. Reddy Labs. Prazosin and Terazosin were obtained from Arti laboratories and Nosch Pharma, respectively. Methanol, acetonitrile, and water (HPLC-grade) were purchased from Merck Ltd (Mumbai). Other reagents were all of analytical grade. Doxacard 2 mg batch no. A11251 (Cipla ltd.), Terapress 2 mg batch no. DM1160 (Intas Pharmaceuticals), and Prazopress XL 5 mg batch no. JKK1147 (Sun Pharmaceuticals) were purchased from a local pharmacy market.

### Chromatography apparatus and conditions

The Waters HPLC system was equipped with the Empower software and composed of the Quadrupole 600E gradient pump with a four channel multisolvent delivery system, an online Waters In-line degasser AF, a column oven model HCO-O2 (PCI analyst), and a 2998 PDA detector. All separations were carried out on a new Kromasil C_18_ column (250 × 4.6 mm, 5.0 μm) from Agilent Technologies and was used for development of the HPLC study. Wavelength selection (254 nm) is based on a UV overlapping graph [38] presented in our earlier publication.

### Validation of stability indicating method

#### Specificity

To prove specificity of the method, the sample was kept under different stressed conditions. The stress study under acidic hydrolysis follows: the drug solution in 0.1N HCl was exposed at 80 °C for 10 h; alkaline hydrolysis: the drug solution in 0.1N NaOH at 80 °C for 20 min. For oxidative stress conditions, drug solutions in 3 and 30% H_2_O_2_ were stored at room temperature for 6 and 24 h, respectively. For photolytic degradation, samples were kept under sunlight at ambient temperature for 3 days. For thermal degradation, conditions were 60 °C for 3 days.

#### Linearity

Suitable aliquots of the standard solution containing terazosin, prazosin, and alfuzosin (each 1 mg/ml) in methanol were diluted in methanol to produce 2, 20, 50, 100, 200, 300, 400 and 500 μg/ml dilutions in separate 10 ml volumetric flasks. Solutions were filtered by a 0.45 μ filter before injecting into the HPLC system. The data of peak area versus drug concentration was treated by linear least square regression analysis.

#### Precision

For repeatability, a solution of 50 μg/ml was injected six times and a relatively standard solution was calculated. Intraday and interday dilutions of 20, 50 and 100 μg/ml were injected three times in the same day and in three consecutive days, respectively.

#### Accuracy

Suitable aliquots of the standard stock solution were further diluted to prepare 20, 50, and 100 μg/ml solutions that were previously spiked with commonly used excipients of tablet formulations like starch, lactose, magnesium stearate, and an opacifier such as titanium dioxide. The recovery was calculated by comparison with unspiked samples.

#### Limit of detection and limit of quantitation

In order to estimate the limit of detection and the limit of the quantitation standard deviation of the intercept obtained from the preparation, a calibration curve was used. Calculations were performed for all drugs by using following formula.

$$\begin{array}{l} \text{LOD}=\frac{3.3\times \text{SD}}{\text{Slope}}\\ \text{LOQ}=\frac{10\times \text{SD}}{\text{Slope}} \end{array}$$

Where, SD is the standard deviation of the intercept

#### Stability of analyte

A solution of 50 μg/ml was analyzed at intervals of 0, 24, and 48 hrs, storing the samples at room temperature. Relative standard deviations of data gave an estimate of the stability of the analyte.

#### System suitability

A solution of 100 μg/ml was injected six times and the variation in number of theoretical plates, peak area, tailing factor, and capacity factor were calculated.

## Results and discussion

### Optimization of the mobile phase

Initially, various proportions of methanol and water were used as the mobile phase under isocratic conditions, but separation was not adequate. Use of diethylamine with an acetonitrile methanol mixture was found to improve the resolution between prazosin and terazosin. Ammonium acetate solution was found to improve the tailing of doxazosin. Thus, it was found that the mobile phase consisting of A: CAN–diethylamine (0.05 ml), B: methanol, and C: 10 mM Ammonium acetate, gives good separation between these drugs. The gradient condition of the mobile phase (A:B:C) was: 60:40:0:0 for 8 min, 60:20:20:0 for 1 min, 60:0:40:0 for 5 min, and a further 60:40:0:0 gradient for 1 min for system equilibration. The wavelength selected was 254 nm. The retention times of terazosin, prazosin, and doxazosin were found to be 2.472, 6.0, and 10.22, respectively.

### Specificity

**Fig. 2:**
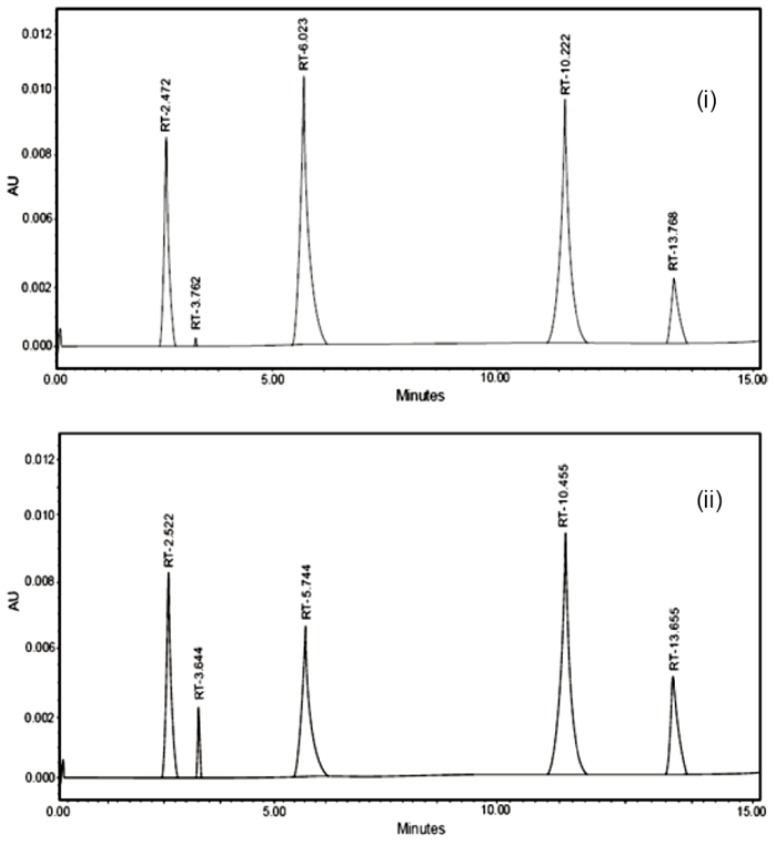
Chromatogram under different stress conditions. (i) Acidic hydrolysis (ii) Alkaline hydrolysis

**Fig. 3:**
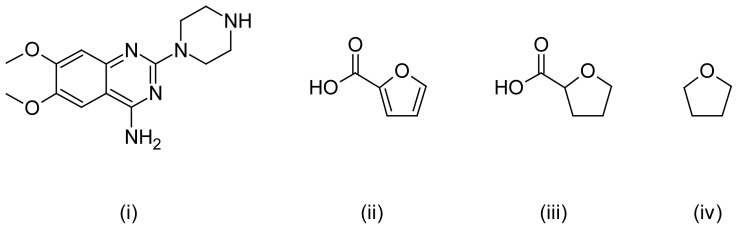
Probable degradants: (i) 6,7-dimethoxy-2-(piperazin-1-yl)quinazolin-4-amine, (ii) 2-furoic acid, (iii) tetrahydrofuroic acid, (iv) tetrahydrofuran

Samples were subjected to acidic, alkaline, oxidative, photolytic, and thermal stress conditions. Chromatograms obtained under acidic and alkaline hydrolysis conditions were reported to the formation of 2-piperazinyl-6,7-dimethoxy-4-aminoquinazoline by Bakshi et al^40^. Another product, 2-furoic acid, with retention time around 3.6 min was formed during degradation of prazosin under acidic and alkaline conditions. This peak is ascribed to 2-furoic acid, the leaving group for this drug. Terazosin forms tetrahydrofuroic acid, which decomposes further to tetrahydrofuran and carbon dioxide [37]. No degradation was found under oxidative degradation and thermal stress conditions. However, degradation was observed in photolytic degradation, resulting in the formation of a number of degradants. The resolution of all the degradants was found to be more than 1.5, which proves that this method is specific in nature. Figure 2 and 3 represent chromatograms under different stress conditions and potential degradants respectively.

### Linearity

**Fig. 4:**
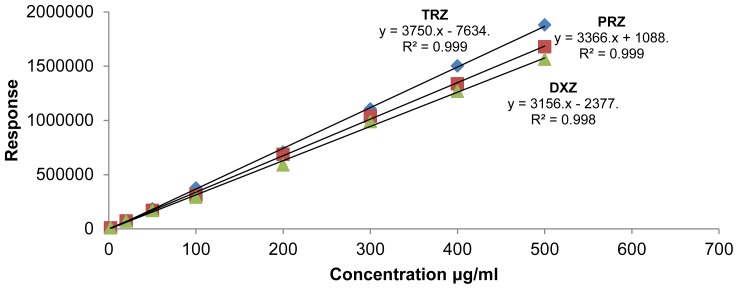
Calibration curve of Terazosin, Prazosin and Doxazosin by proposed method

The regression equations for terazosin, prazosin, and doxazosin were found to be y = 3750 × − 7634, y = 3366 × + 1088, and y = 3156 × − 2377, respectively. Correlation coefficients were found to be 0.999, 0.999, and 0.998 for terazosin, prazosin, and doxazosin, respectively, which proves that the calibration curve is sufficiently linear. Figure 4 represents the calibration curve obtained by the proposed method.

### Precision

Results of repeatability, interday, and intraday for terazosin, prazosin, and doxazosin were presented under Table 1. The overall relative standard deviation was found to be less than 1, which proves that the method is sufficiently precise to continue this study.

### Accuracy

Recovery studies were performed by spiking commonly used excipients of tablet formulations with a known concentration of drugs. Results are shown under Table 2. The percentage recovery found was between 100.14–98.92, and the established method can be accurately depicted in tablet formulations.

### Limit of detection and quantitation

The limits of detection for terazosin, prazosin, and doxazosin were found to be 0.065, 0.033, and 0.109 μg/ml respectively. Similarly, the limits of quantitation were found to be 0.197, 0.102, and 0.332 μg/ml, respectively.

### Stability of analytes

There is no appreciable loss of any drugs found up to 48 hours. This study proves the method is sufficiently stable when used within this time period.

### System suitability

Data of the system suitability parameter is presented under Table 3.

### Analysis of formulations

The tablet powder equivalent to 5, 2 and 2 mg PRZ, TRZ, and DXZ, respectively, were transferred to a 10 ml volumetric flask and diluted with methanol to about 50 ml. The solution was ultrasonicated for 15 minutes and diluted up to the mark with methanol. The solutions were then carefully filtered with a 0.45 μ membrane filter before injecting into HPLC system. The result of the analysis of formulations is mentioned under Table 4.

## Conclusion

The current study is validated in terms of specificity, accuracy, precision, linearity, LOD, and LOQ. While the evaluation of these parameters is found to fulfill the desirable minimum criteria, it also proves that methods can be used for the quality control of these drugs in the pharmaceutical industry. Specificity was judged by keeping samples under different stress conditions. All of the degradants were separated by the parent drug peaks. This method was also found to be sufficiently linear, precise, and accurate under the prescribed conditions. Common excipients of tablet formulations are not found to interfere during the extraction of drugs in matrices as mentioned under the recovery studies. However, the application of the proposed method in biological samples is suspicious, which may be due to insufficient sensitivity, but the mobile phase can be used to develop bioanalytical methods in the case if peaks of blank biological samples did not interfere. It is evident that drugs with similar structure are difficult to separate, thus the presented study can be utilized to save time in separation of terazosin, prazosin, and doxazosin in the pharmaceutical industry. Moreover, any one of the drugs can be used as the internal standard for the determination of another drug. The proposed study can be utilized for the determination of the three structurally similar drugs prazosin, terazosin, and doxazosin in their respective real samples.

## Figures and Tables

**Tab. 1:** Data for precision study by the proposed method

Drug	Precision type	Concentr. (μg/ml)	Mean (Area)	SD	RSD
TRZ	Repeatability	50	182397.5	121.765	0.066
		20,	75543.3,	232,	0.307,
	Intraday	50,	182514.6,	354.78,	0.194,
		100	375011	570.57	0.152
		20,	75585,	313.46,	0.415,
	Interday	50,	182487.3,	458.37,	0.25,
		100	374959.3	699.78	0.187

PRZ	Repeatability	50	170221.1	178.22	0.104
		20,	72437.3,	376.13,	0.52,
	Intraday	50,	170480.6,	850.08,	0.499,
		100	310207	863.42	0.278
		20,	72410.67,	418.1,	0.577,
	Interday	50,	170477.7,	960.07,	0.563,
		100	309651.7	1137.086	0.367

DXZ	Repeatability	50	167513.1	353.25	0.211
		20,	70355.3,	463.3,	0.658,
	Intraday	50,	167278,	922.78,	0.551,
		100	290640.3	1149.48	0.395
		20,	70599.67,	561.26,	0.794,
	Interday	50,	167299.7,	1039.549,	0.621,
		100	290884.3	1278.941	0.44

**Tab. 2:** Data for recovery studies by the proposed method

Drug	Concentr. level	Before Spiking		After Spiking		Recovery (%)
	
		Area found	Concentr. (μg/ml)	Area found	Concentr. (μg/ml)	
TRZ	20	75122	22.07	75212	22.09	100.11
	50	182655	50.74	182544	50.71	99.94
	100	373666	101.7	374211	101.82	100.14

PRZ	20	72833	21.31	72444	21.20	99.46
	50	169897	50.15	169166	49.93	99.57
	100	358612	106.22	358121	106.07	99.86

DXZ	20	70034	22.94	69756	22.85	99.62
	50	167856	53.94	167022	53.67	99.51
	100	304346	97.19	301023	96.13	98.92

**Tab. 3:** Data for system suitability of the proposed method

Drug	Theoretical Plates (n=6)			Tailing Factor (n=6)			Capacity Factor (n=6)		
	SD	Mean	R.S.D	SD	Mean	R.S.D	SD	Mean	R.S.D
TRZ	7.11	7029.16	0.101	0.00548	1.055	0.519	0.0131	2.3575	0.558
PRZ	12.72	6034.16	0.210	0.00837	1.105	0.757	0.0336	5.569	0.604
DXZ	24.77	5930.16	0.417	0.00837	1.085	0.771	0.0843	9.535	0.884

Percentage relative standard deviation in each case found to be less than 1.

**Tab. 4:** Result of analysis of formulations by the proposed method.

Name of formulation	Drug	Assay±SD(n=3)	Percentage
Doxacard (Cipla)	Doxazosin	1.97±0.010	98.5%
Terapress (Intas)	Terazosin	1.96±0.011	97.8%
Prazopress (Sun Pharma)	Prazosin	4.93±0.057	98.7%
